# Evaluation of the Effects of Retro-Cavity Preconditioning with or Without Ethylenediaminetetraacetic Acid on Root Surface pH and Dislodgement Resistance of NeoMTA2 and Mineral Trioxide Aggregate Flow Retro-Fills: An Ex Vivo Investigation

**DOI:** 10.3390/jfb16010003

**Published:** 2024-12-24

**Authors:** Sedigheh Khedmat, Seyyed Ali Abaee, Hadi Assadian, Antonio Signore, Stefano Benedicenti

**Affiliations:** 1 Department of Endodontics, School of Dentistry, Tehran University of Medical Sciences (TUMS), Tehran 14399-55991, Irans.a.abaee73@gmail.com (S.A.A.); 2 Therapeutic Dentistry Department, Institute of Dentistry, I.M. Sechenov First Moscow State Medical University, Trubetskaya Str. 8, b. 2, 119992 Moscow, Russia; 3 Department of Surgical Sciences and Integrated Diagnostics, University of Genoa, Viale Benedetto XV, 6, 16132 Genoa, Italy

**Keywords:** bond strength, calcium silicate cement, dentin, EDTA, root canal

## Abstract

**Background:** The aim of this study was to investigate the effects of retro-cavity preconditioning with or without 17% ethylenediaminetetraacetic acid (EDTA) solution on root surface pH as well as dislodgement resistance of NeoMTA2 and MTA Flow retro-fills. **Methods:** Forty-eight single-rooted human incisors were selected. After completion of endodontic treatment, root-end resections were performed, and retro-cavities were prepared. The samples were randomly divided into two groups of A and B (*n* = 24 each). In group A, retro-cavities were preconditioned with 2.5% NaOCl, followed by 17% EDTA solution, whereas in group B, preconditioning was performed using 2.5% NaOCl before final irrigation with normal saline. Samples in each group were randomly subdivided into two subgroups of 1 and 2. Retro-fillings in the A1 and B1 subgroups were performed with MTA Flow, and in the A2 and B2 subgroups, they were performed with NeoMTA2. Root surface pH was measured in each sample at three different stages: before preparation of retro-cavities (pH0), after retro-cavity preconditioning (pH1), and three days after retro-filling (pH2). Subsequently, the push-out bond strength (PBS) of the retro-filling materials was measured by a universal testing machine, and their failure modes were visualized under 64× magnification. **Results:** Preconditioning with EDTA caused a significant increase in PBS for both NeoMTA2 and MTA Flow (*p* < 0.001). There was no significant difference between the average bond strength of MTA Flow and Neo MTA2 (*p* = 0.271). There was a significant increase in the average pH2 compared to pH1 and pH0 across all groups (*p* < 0.001). Specifically, the use of EDTA led to a notable increase in the average pH2 in the MTA Flow group compared to the Neo MTA2 group (*p* = 0.027). Groups preconditioned with EDTA more frequently indicated a cohesive failure mode. **Conclusions:** The use of EDTA significantly increased the push-out bond strength of retro-fill materials to dentin. However, it did not prevent the ultimate alkalinity of retro-filled cavities.

## 1. Introduction

Periradicular surgery is indicated to remove causative irritants when nonsurgical endodontic (re-)treatments result in persistent disease [1]. It involves several steps, including retro-filling the resected and prepared root end cavity with an appropriate material [2]. In order to have long-term success, mineral trioxide aggregate (MTA) has been widely accepted to be used as the retro-filling material due to its sealing ability, biocompatibility, hard-tissue stimulation, and antibacterial properties [3,4,5]. To overcome some disadvantages of MTA, such as long setting time, poor handling properties, low resistance to compression and bending, and tooth discoloration potential, a few changes have been made to its original formulation [6]. For example, Neo MTA 2 (NuSmile Avalon Biomed, Bradenton, FL, USA) was recently introduced with fine powder particles and tantalum oxide as the radiopacifier. It is mixed with a gel in a desired consistency to provide users with improved handling properties [7]. Another example is MTA Flow (Ultra dent Products Inc., South Jordan, UT, USA), which consists of a water-soluble silicone-based gel and a gray powder containing bismuth oxide, tricalcium, and dicalcium silicate. It can be prepared in different consistencies by changing the powder-to-gel ratio, thereby providing clinicians with ease of material application and placement for different endodontic treatments [8]. In addition to similar physico-chemical properties, such as low solubility, acceptable radiopacity, and comparable ability to deposit calcium phosphate, a similar alkalinizing capability of MTA Flow has also been reported in comparison with MTA Angelus after being soaked in simulated body fluid [9].

It is preferable for a retro-filling material to have a strong bond at the material–dentin interface to resist against dislodging forces during function [10]. It is believed that the bond strength of materials to root dentin is in line with the material sealing ability [11]. The push-out bond strength (PBS) test is considered as one of the most well-recognized laboratory assessments in evaluating the bond strength between root filling material and dentin [12,13]. Although the main advantage of the PBS test over other bond testing approaches is evaluating the material bond strength while being surrounded by dentin, there is no consensus on whether the results of this assessment are clinically applicable [2].

Dentin conditioning with ethylenediaminetetraacetic acid (EDTA) has been widely advocated in endodontics. It has been demonstrated that EDTA, as a chelating agent, is capable of removing inorganic components in the dentin structure as well as the smear layer created following dentin preparation. Promoting cyto-differentiation and tissue formation, as well as provoking growth factor release throughout the root canal, dentin has been pointed out as another advantage of EDTA application in endodontics [14]. On the other hand, an alkaline pH following the use of calcium silicate cements (CSCs) is required to eliminate endodontic pathogens. It also can favor apatite nucleation, the release of alkaline phosphatase, and bone morphogenetic protein 2, which are involved in periapical healing [15,16].

Since limited information is available concerning the physico-chemical properties of NeoMTA2 and MTA Flow, specifically when used as retro-fill materials, and considering that these recently introduced materials claim to have improved handling properties, the present study aimed to evaluate the dislodgement resistance of these two materials used as retro-fills in an ex vivo model, as well as the effects of retro-cavity preconditioning with or without EDTA on root surface pH changes.

## 2. Materials and Methods

This ex vivo investigation was conducted according to the Preferred Reporting Items for Laboratory studies in Endodontology (PRILE) 2021 guidelines [17] and was approved by the Research Ethics Committee of the School of Dentistry, Tehran University of Medical Sciences (IR.TUMS.Dentistry.Res.1401070). It was performed on human extracted maxillary incisor teeth. The sample size estimation was conducted based on the study by Gokturk et al. [18], using Power Analysis and Sample Size (PASS) software (version 11.0.7; PASS, NCSS, LLC) with an alpha error probability of 0.05. For retro-fill material, treated as a dichotomous variable, an effect size of 0.667 was applied. Similarly, an effect size of 0.417 was used for the dichotomous variable of preconditioning, with the dependent variable being root surface pH. The statistical power was 0.995 for the first and 0.81 for the second variable. Therefore, the sample size for each group was determined to be *n* = 12.

### 2.1. Sample Selection and Preparation

In this study, a total of 48 caries-free and resorption-free teeth, extracted for periodontal reasons, were included. Teeth with straight roots and type I root canal anatomy based on Vertucci’s classification were selected, while those with cracks, fractures, or developmental anomalies were excluded. To ensure reproducibility, several assessments were conducted to detect cracked and fractured teeth before or during the investigation. Clinical visualization was performed using a dental explorer and magnification under a dental operating microscope to exclude teeth with visible cracks or fractures. Radiographic analysis, including the evaluation of preoperative periapical radiographs in the buccolingual and mesiodistal directions, was used to exclude teeth with fractures extending to the root surface, including vertical root fractures. Additionally, transillumination with fiber optic light helped reveal and exclude teeth with cracks not visible to the naked eye. These radiographs also confirmed Vertucci’s type I root canal configuration, apical maturity, and the absence of previous endodontic treatments. The soft tissues on the root surfaces were removed with a scaler (Universal Sickle Scaler, Nordent, Solingen, Germany), and the teeth were kept in 0.5% Chloramine-T solution (Merck, Darmstadt, Germany) for disinfection for 24 h.

Initially, an access cavity was prepared on each tooth using a high-speed fissure bur (Livingstone, Seoul, Republic of Korea) under water and air spray. Then, a #10 K-File (Micro-Mega, Besançon, France) was inserted into the root canal and extruded from the apical foramen. As soon as the file tip was seen from the root end, a one-millimeter short measurement was considered as the working length for the entire root canal preparation and obturation procedures. Root canal preparation was performed using the Gold Denco rotary system (Shenzhen Denco Medical Co., Shenzhen, China) up to the #F3 rotary file according to the manufacturer’s recommendations. Root canals were irrigated with 2.5% NaOCl solution (Morvabon Co., Tehran, Iran) between each file use. The teeth were obturated using cold lateral compaction with 0.02 taper gutta-percha points (Data Co., Beijing, China) and AH 26 sealer (Dentsply DeTrey GmbH, Konstanz, Germany). Afterward, the teeth were incubated at 37 °C for 24 h under 100% humidity. Subsequently, root-end resection was performed on each tooth root at a 3 mm distance from the apex using a carbide fissure bur (Jota AG, Rüthi, Switzerland) under water spray, and a 3 mm deep retro-cavity was prepared using diamond-coated retro-tips powered by an ultrasonic device (Ultra Mint Pro, Eighteenth Co., Changzhou, China) using E (endodontic) mode at the power of #3 under water spray.

### 2.2. Preparation and Preconditioning of the Retro-Cavities

The samples were randomly divided into two groups of A and B, each containing 24 samples using simple randomization aided by www.randomization.com (accessed on 21 February 2022). In group A, retro-cavities were preconditioned with 5 mL of 2.5% NaOCl, followed by 5 mL of 17% EDTA solution (Morvabon Co., Tehran, Iran) for 1 min for all solutions. In group B, the retro-cavities were preconditioned using 2.5% NaOCl and irrigated by normal saline solutions using the same volumes and times. All specimens were rinsed thoroughly with 5 mL of normal saline (Daroo-Pakhsh Co., Tehran, Iran) for 1 min to eliminate the possible effects of preconditioning solutions on retro-fill materials.

The samples in each group were randomly divided by the same method mentioned above into two subgroups of 1 and 2. Retro-fillings in the A1 and B1 subgroups were performed with MTA Flow (Ultradent Products Inc., South Jordan, UT, USA) and in the A2 and B2 subgroups, with NeoMTA2 (NuSmile Avalon Biomed, Bradenton, FL, USA) (See Table 1). In all subgroups, the retro-filling materials inside the cavities were 3 mm thick. The retro-cavities were completely dried prior to retro-fillings, and each material was prepared and placed according to the manufacturer’s recommendation. Eventually, the surface of each retro-fill material was covered with a wet cotton pellet and placed in a specified falcon.

The teeth were subsequently incubated at 37 °C in 100% humidity for 7 days to ensure completion of the retro-fill setting reactions.

### 2.3. Measurement of pH

The measurement of root surface pH was performed by a blinded operator who was unaware of the specific procedures and materials used. A digital pH meter pen (Sentek Co., Braintree, UK) was utilized for these measurements within the specified falcon for each sample. The values displayed on the device monitor were recorded as the sample’s pH. Prior to each measurement, the device was calibrated according to the manufacturer’s recommendations using a neutral substance with a known pH of 7. The pH measurement for each sample was conducted at three different stages:

A. At the beginning of this study, before the preparation of retro-cavities (recorded as the baseline or pH0);
B. Immediately after retro-cavity preconditioning (recorded as pH1);
C. Three days after placing the retro-fill materials (recorded as pH2).

### 2.4. Push-Out Bond Strength (PBS) Test

After one week, the setting of the retro-filling materials was confirmed by a size #B endodontic spreader (Dentsply-Maillefer, Tulsa, OK, USA). The samples were placed in a mold filled with self-curing acrylic resin (Beta Dent, Tehran, Iran). The samples were buried from the coronal region so that the apical end of the cavity was tangential to the distal edge of the acrylic resin. After the complete setting of the acrylic molds, the samples were removed from the mold to prepare one-millimeter disks at a one-millimeter distance coronal to the resected apex. Initially, the samples, which were held in cylinders, were fixed horizontally on the stand of the cutting machine using glue (Super glue, Razi, Iran). Then, the samples were horizontally fixed from the apical area and were cut with a diamond disk under water and air spray at a slow speed by a cutting machine (Pars Mechatronic, Tehran, Iran). The samples were visualized under 3.5× magnification to rule out any fractures or defects. Then, the surface roughness of each sample was smoothed out using soft sandpaper. After preparing all the disks, the push-out bond strength was measured by a blinded operator using a universal testing machine (UTM) (Santam Co., Tehran, Iran).

In order to perform the push-out bond strength (PBS) test, the prepared disks were fixed with a piece of wax in the universal testing machine, and a plunger, 0.7 mm in diameter, was fit to the retro-filled portion at the center of the disk to apply push-out force at the speed of 0.5 mm per minute until dislodgement occurred. Meanwhile, a diagram related to the application of force was drawn by SCM-3000 software (v. 14.7.9, Microtest; Madrid, Spain). Therefore, within the force diagram drawn by the software, which had an upward trend, an immediate drop was observed, which indicated failure. The maximum force required to dislodge the retro-filling material was recorded in Mega Pascals (Mpa).

Eventually, the sections were examined under 64× magnification using a scanning stereomicroscope (Olympus, Japan) in order to investigate the state of bond failure. Then, a photomicrograph of each disk sample was taken to visualize the type of failure.

**Figure 1:**
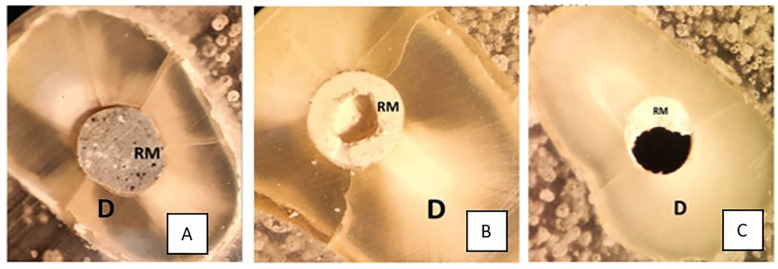
Stereomicroscopic images of the types of bond failures: (**A**) adhesive failure, (**B**) cohesive failure, and (**C**) mixed failure.

Types of bond failure situations were classified as follows:

(a) Adhesive failure, which occurred between the retro-filling material and the dentin. (Figure 1A);
(b) Cohesive failure, which occurred within the retro-filling material (Figure 1B);
(c) Mixed failure, which encompassed both the abovementioned failure types (Figure 1C).

### 2.5. Statistical Analysis

The Kolmogorov–Smirnov test was used to assess the normal distribution of the data, and the two-way ANOVA test was used to determine the effect of preconditioning on the PBS of the retro-filling material as well as the root surface pH values. A *p*-value of less than 0.05 was considered statistically significant.

## 3. Results

This investigation evaluated the effects of the use of 17%EDTA as a preconditioner on the dislocation resistance of Neo MTA2 and MTA Flow retro-fills, as well as root surface pH changes after 3 days.

### 3.1. Push-Out Bond Strength (PBS)

**Figure 2:**
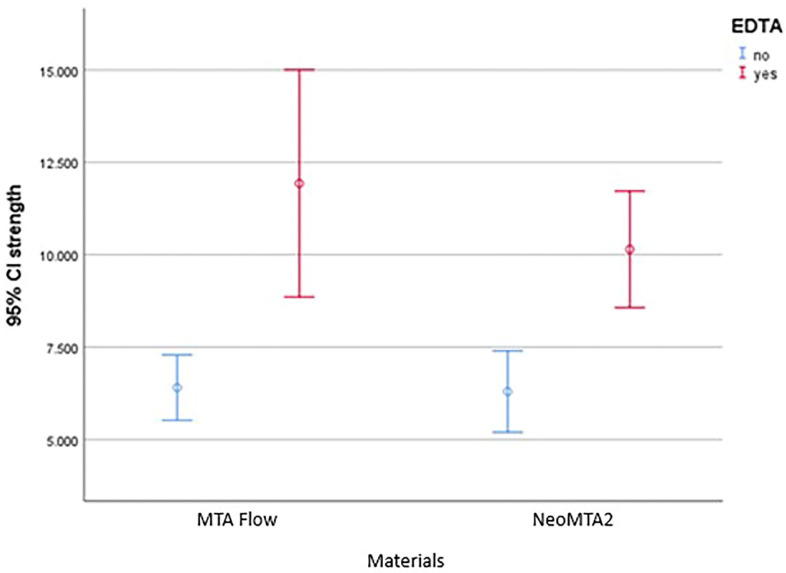
Push-out bond strength of experimental materials with 95% confidence interval, with or without preconditioning with EDTA (no: without preconditioning with EDTA; yes: conditioning with EDTA).

As shown in Figure 2, preconditioning the retro-cavities with 17%EDTA caused a significant increase in the PBS of the samples (*p* < 0.001). The results showed that there was not a statistically significant difference between the A1 and A2 subgroups (*p* = 0.271).

### 3.2. pH Changes

#### 3.2.1. pH1 (After Retro-Cavity Preconditioning)

The results showed that there was a significant decrease in average root surface pH1 in group A compared with group B (*p* < 0.001). There was not any significant difference between the average pH1 in the A1 and A2 subgroups (*p* = 0.212).

#### 3.2.2. pH2 (3 Days After Placing the Retro-Filling Materials)

The average pH2 showed significantly lower values in the A2 subgroup compared to the B2 subgroup (*p* = 0.011).

Additionally, while comparing different retro-fill materials, higher pH2 values were observed in the A1 subgroup compared to the A2 subgroup (*p* = 0.027). However, there was no significant difference between the B1 and B2 subgroups (*p* = 0.141). There was not any significant difference between the average pH2 values in the A1 and B1 subgroups (*p* = 0.511).

#### 3.2.3. Comparison of pH1 and pH2

**Figure 3:**
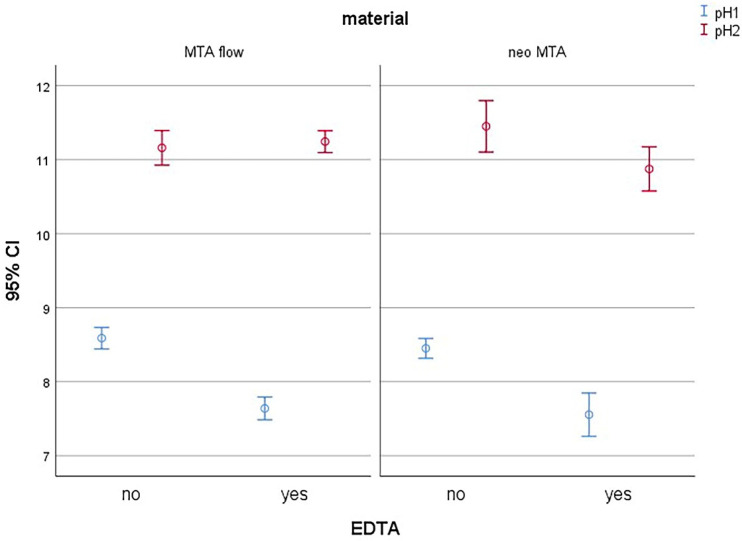
Average pH1 and pH2 measurements before and after the use of retro-fill materials with or without preconditioning with EDTA.

As shown in Figure 3, regardless of the type of retro-filling material used or whether or not preconditioning with 17%EDTA was performed, there was a significant increase in the average pH2 in comparison with pH1 (*p* < 0.001).

#### 3.2.4. Comparison of pH1 and pH2 with pH0

The average values for pH1 were significantly higher than those for pH0 in the B1 and B2 subgroups (*p* < 0.001). However, no significant difference between pH0 and pH1 values was observed between the A1 and A2 subgroups. As demonstrated in Table 2, there was a significant increase in pH2 values compared with pH0 in all subgroups (*p* < 0.001).

### 3.3. Modes of Failure

**Figure 4:**
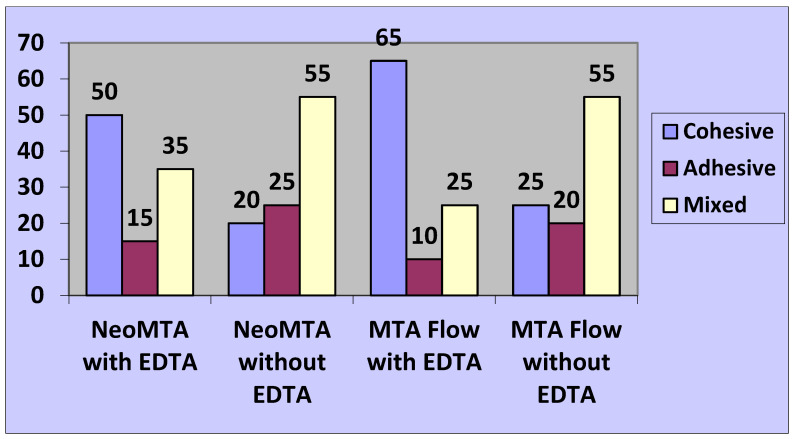
Percentage of failure types observed across various subgroups.

The prevalent form of bond failure observed was cohesive within the A1 and A2 subgroups while demonstrating a mixed failure type within the B1 and B2 subgroups. (See Figure 4 for details).

## 4. Discussion

Providing adequate apical seals against the trafficking of bacteria and their byproducts is among the most important factors influencing the long-term success of periradicular surgery [1]. Adequate frictional or micromechanical bond strength between calcium silicate cements (CSCs) used as retro-fillings and radicular dentin is advantageous in providing a sustainably impervious interface [2,12,13]. In this realm, the dislodgement resistance of a retro-filling material can be typically evaluated using a PBS test [13]. It has been documented that PBS can correspond with clinical situations in which forces are applied parallel to the long axis of the tooth, creating a shear force at the material–dentin interface [19,20]. The PBS test has been considered by a number of investigators as an efficient, reliable, and practical method for evaluating the bond strength of a (retro-)filling material to root canal dentin. Several factors are influential in the results of the PBS of an endodontic (retro-)filling material, such as the irrigants used prior to the material application, containment of the retro-fill material within radicular dentin (increased C-factor), the presence or absence of the smear layer, and the tubular size and density in surrounding dentin [10,21,22]. In this study, Neo MTA2 and MTA Flow were the CSCs used as retro-fillings due to their recent introduction as well as improved handling and structural properties. To the best of the authors’ knowledge, limited studies were found in the available literature to evaluate their physico-chemical properties. Therefore, this study aimed to investigate the effects of retro-cavity preconditioning with or without EDTA on the dislodgement resistance and failure modes of NeoMTA2 and MTA Flow retro-fills, as well as their impact on root surface alkalinity in an ex vivo model.

The use of a 17% EDTA solution has been advocated by some authors in preconditioning retro-cavities [23,24]. EDTA is a weak acidic solution with a pH ranging from 4 to 6. It is used in endodontics to help remove the organic components of the smear layer. Some authors have supported the use of EDTA in endodontic and periodontal surgeries [25,26]. Acting as an anti-oxidant, EDTA is capable of removing the so-called “oxygen-rich layer”, which can, in turn, improve the interaction between CSCs used as retro-fillings and radicular dentin [27]. EDTA performs as a chelating agent that has an affinity to divalent cations, e.g., calcium ions, and can make them into soluble chelates. Its chelating effect on the bacterial cell wall, which results in more susceptibility to antibiotics and antibacterial agents, can be responsible for its bactericidal effect [28]. It is applied to remove inorganic components of the smear layer and is able to release growth factors from dentin and stimulate cell differentiation [14]. However, compromised dentin structure due to microscopic erosions, as well as decreased bond strength and microhardness of CSCs, has been reported following the use of EDTA due to its interference with CSC hydration [14,29]. Nevertheless, in the current investigation, preconditioning the retro-cavities with EDTA resulted in an increase in the bond strength of NeoMTA2 and MTA Flow with the dentin wall. This finding is in line with those of Gokturk et al., who similarly stated that a significant increase in dislocation resistance of MTA Flow and MTA Angelus was found following the application of 17% EDTA compared to chitosan-based silver nanoparticles (AgNP–chitosan) and maleic acid [18]. Consistently, Valencia et al. stated that 17% EDTA was able to improve the PBS of calcium silicate cement to dentin when used as a retro-filling material [30]. This was also supported by Ballal et al. [20], who reported that the use of 17% EDTA as an irrigation solution could increase the PBS of Biodentine. Likewise, Anju et al. [31] also showed that the maximal increase in the PBS of NeoMTA Plus was achieved when 17% EDTA was used as the preconditioner. This can be due to the capability of EDTA application following NaOCl irrigation of removing the smear layer, which could aid in forming retro-fill material tags into conditioned dentin, increasing the micromechanical surface area at the bond interface [32,33]. These results are contradictory to the finding of Paulson et al. [34], who concluded that in root canals irrigated with 2.5% sodium hypochlorite and 17% EDTA, lower PBS values were recorded in EDTA-treated groups following the use of Biodentine as the retro-filling. The authors claimed that the acidic nature of EDTA could have detrimental effects on the hydration of the CSCs, which could, in turn, result in impaired bonding capabilities. Additionally, this contradictory result can be related to the difference in dentin structure where the samples were taken from. Contrary to our investigation, the dentin samples in Paulson et al.’s study were taken from the middle third of the root, which has different tubular and structural patterns compared with the apical dentin used in the current investigation. In addition, Gade et al. [35] demonstrated that the use of 17% EDTA could compromise the PBS of CSCs to dentin. This might be related to the immersion of root sections in sodium hypochlorite for 5 min and in 17% EDTA for 30 min, which could deteriorate the dentin structure for further assessment. EDTA effectively removes the smear layer and exposes dentinal tubules, facilitating improved penetration and adhesion of root-filling materials [36]. This stronger adhesion reduces the risk of microleakage, a common cause of treatment failure [37]. Research suggests that using EDTA as a final flush during retro-cavity preparation enhances the durability and strength of the seal, improving the success rate of endodontic surgeries [20]. Materials with higher bond strength to root dentin and deeper penetration into dentinal tubules exhibit superior sealing properties, emphasizing the importance of achieving a strong bond between the retro-filling material and the root dentin [38]. Treating retro-cavities with EDTA can significantly enhance the push-out bond strength of retro-fillings. Therefore, by minimizing the risk of microleakage and reinfection, EDTA-treated retro-cavities can help promote healing and reduce treatment failure.

In this study, higher PBS was associated with a more frequent cohesive failure of the materials. This could indicate that dislodgement forces applied to the tested materials were able to cause a more frequent material disintegration rather than material separation from the root canal walls. This can be supported by the findings of some authors who pointed out that a strong bond is formed between CSCs and dentin via the formation of interfacial tag-like structures during biomineralization through a diffusion-controlled reaction [39]. Confocal laser as well as scanning electron microscopic images revealed that the flowable consistency of CSCs could permit intra-tubular penetration of the cements. In addition, a structurally altered inter-tubular dentin subjacent to the investigated CSCs was visualized at the cement–dentin interface referred to as the “mineral infiltration zone”, which conceptually resembled the dentin–adhesive interface [40]. The type of failure for each retro-filling material under dislodgement forces can be closely related to the amount of PBS [34]. In the present study, the subgroups preconditioned with EDTA showed higher PBS records, indicating the cohesive failure mode as the most common feature encountered. This finding can endorse stronger bonds between retro-fill materials and EDTA-treated dentin surfaces. However, a mixed type of failure mode was observed in non-EDTA-treated subgroups as the most frequent finding. The occurrence of different types of bond failures within the same subgroups can likely be attributed to the assumption that the failure threshold among the samples does not vary significantly. This implies that even when the same retro-filling material or preconditioning regimen is used, multiple types of failures may still be observed within each subgroup. However, when making comparisons across different experimental groups, it is important to focus on the most prevalent type of failure, as this provides a clearer basis for analysis and interpretation. To further validate this hypothesis, future studies with larger sample sizes are strongly recommended. Larger datasets would allow for a more comprehensive evaluation of whether differences in failure thresholds exist and how they influence the observed types of bond failure within and across subgroups.

An alkaline pH following the release of hydroxyl and calcium ions from CSCs during endodontic surgical interventions favors the elimination of the remaining pathogenic microorganisms via reacting carbon dioxide as well as fostering apatite nucleation and increased hard-tissue formation via upregulating alkaline phosphatase and bone morphogenetic protein 2 (BMP2) which, in turn, promotes sealing ability of CSCs [41,42,43,44]. In this study, NeoMTA2 and MTA Flow were able to alkalinize the environment similar to other CSCs.

The present study showed that 17% EDTA used to precondition the retro-cavities could initially decrease environmental pH, but it did not interfere with the secondary alkalinity induced by the CSCs after 3 days. The present study showed that MTA Flow was more effective than NeoMTA2 in creating a higher pH on the root surface after three days. This finding may probably be due to a more pronounced capability of MTA Flow to release hydroxyl ions to a preconditioned environment in comparison with NeoMTA2, but this needs further evaluation. Since pH differences were not considered statistically significant in the non-preconditioned groups, this might be attributable to a chemical interaction between MTA Flow and EDTA in the presence of dentin. However, this should be corroborated by more detailed investigations. Guimarães et al. [9] demonstrated more alkalinity of MTA Flow compared with MTA Angelus in a similar time interval. The findings of this study reveal that the pH level of NeoMTA2 in retro-cavities treated with 17% EDTA was significantly lower than in those without such treatment. This discrepancy may be due to NeoMTA2’s inability to create an environment that increases pH levels in the presence of 17% EDTA. Nonetheless, this observation warrants further detailed investigation to fully understand the underlying mechanisms. Furthermore, since the antibacterial properties of CSCs are often influenced by changes in environmental pH, it is crucial to investigate whether pretreatment with 17% EDTA affects this antibacterial efficacy. Future studies are essential to ensure that CSCs used in EDTA-treated retro-cavities continue to provide the desired antibacterial benefits.

According to the point that the results obtained by laboratory studies cannot be extrapolated to clinical situations, more detailed investigations are recommended to evaluate the effect of preconditioning retro-cavities on the long-term stability of retro-filling materials, as well as their effect on the healing process of the apical periodontitis.

## 5. Conclusions

The use of EDTA significantly increased the push-out bond strength of NeoMTA2 and MTA Flow to dentin. However, it did not prevent the ultimate alkalinity of retro-filled cavities in 3 days.

## Figures and Tables

**Table 1:** Experimental groups categorized based on their preconditioning and retro-fillings.

Experimental Group		Preconditioning	Retro-Filling
A (*n* = 24)	A1 (*n* = 12)	2.5% NaOCl + 17% EDTA + Normal saline	MTA Flow
	A2 (*n* = 12)	2.5% NaOCl + 17% EDTA+ Normal saline	Neo MTA2
B (*n* = 24)	B1 (*n* = 12)	2.5% NaOCl + Normal saline	MTA Flow
	B2 (*n* = 12)	2.5% NaOCl + Normal saline	Neo MTA2

**Table 2:** Comparison of pH1 and pH2 with the baseline pH (=7.5) in experimental groups with or without preconditioning with EDTA.

Material	Preconditioning with EDTA	pH2	*p*	pH1	*p*
MTA Flow	No	11.16 ± 0.37	*p* < 0.001	8.59 ± 0.23	*p* < 0.001
	Yes	11.24 ± 0.23	*p* < 0.001	7.64 ± 0.24	*p* = 0.073
Neo MTA	No	11.45 ± 0.55	*p* < 0.001	8.45 ± 0.21	*p* < 0.001
	Yes	10.87 ± 0.47	*p* < 0.001	7.55 ± 0.46	*p* = 0.692

## Data Availability

The original contributions presented in the study are included in the article, further inquiries can be directed to the corresponding author.

## Abbreviations

EDTA	ethylenediaminetetraacetic acid
MTA	mineral trioxide aggregate
PBS	push-out bond strength
CSC	calcium silicate cement
Mpa	Mega Pascals
